# First Year Medical Students, Personal Handheld Ultrasound Devices, and Introduction of Insonation in Medical Education

**DOI:** 10.5334/aogh.2565

**Published:** 2019-10-15

**Authors:** Mollie Ireson, Simrit Warring, Jose R. Medina-Inojosa, Maria T. O’Malley, Wojciech Pawlina, Nirusha Lachman, Jagat Narula, Anjali Bhagra

**Affiliations:** 1 Mayo Clinic Alix School of Medicine, Mayo Clinic, Rochester, Minnesota, US; 2 Division of Preventive Cardiology, Department of Cardiovascular Medicine, Mayo Clinic, Rochester, Minnesota, US; 3 Department of Anatomy, Mayo Clinic, Rochester, Minnesota, US; 4 Department Cardiology, Mount Sinai St. Luke’s Hospital, Icahn School of Medicine at Mount Sinai, New York, US; 5 Division of General Internal Medicine, Department of Internal Medicine, Mayo Clinic, Rochester, Minnesota, US

## Abstract

**Background:**

Ultrasound education has been provided to students in medical schools within and beyond the United States. A formal experiment with use of personal handheld ultrasound equipment by all first-year medical students has not been reported. Employing insonation (an application of ultrasound) at the personal leisure by medical school freshmen enables self-directed learning throughout the academic year.

**Methods:**

We describe a peer-led ultrasound curriculum with handheld devices. The students’ perceptions were gathered through quarterly Likert-style questionnaires, and the differences in the categories were tested using Analysis of Variance.

**Results:**

The response rate was 58.5% for the first survey (n = 32), 56% (n = 30) for the second survey, and 62.3% (n = 33) for the final survey, respectively, with an average response rate of 58.9%. At the baseline survey, overall agreement was observed for enhancement on performance (62.5%) and interpretation (56.3) of ultrasounds, understanding (68.8%) and learning of anatomy (61.3%), ease (78.1%), comfort (59.4%) and benefit of incorporation of insonation in the medical school curricula (all p-values < 0.001). Neutral response (38.7%) or disagreement (38.7%) was observed when assessing the effect of the integration in medical curriculum on specialty choice (p < 0.01). These trends remained constant over follow-up with the exception that the perceived benefit for integration of insonation into the longitudinal curricula (p < 0.05) increased significantly over time. Majority of disagreement was observed regarding current access to the personal ultrasound devices (38.7%) (p < 0.001).

**Conclusions:**

The introduction of insonation through personal handheld ultrasound devices in the first-year medical school curriculum was received enthusiastically by students, with the majority of respondents finding the devices both easy to use and a valuable aid to improving their understanding of the three-dimensional anatomy.

## Introduction

Medical school curricula across the United States have witnessed an increase in ultrasound education over the past decade [1,2]. This has been concomitant with technological innovations which have brought the traditional ultrasound unit to a size similar to digital tablets and smartphones. Health care facilities around the nation have seen an increase in physicians and providers utilizing handheld ultrasound devices at the bedside, thus promoting the postulation that the handheld ultrasound would find the same status in medical education as has been held by a stethoscope [3]. It has been proposed that the use of a handheld ultrasound, or insonation, will become a fifth pillar of the bedside examination in addition to the inspection, palpation, percussion and auscultation [3]. Although the need to prepare physicians adequately for such technological advances has been a concern for the medical education programs, various surveys have confirmed that teaching medical students to use handheld ultrasound is feasible, increases traditional physical examination skills [4] and helps improve diagnostic accuracy [5]. Even with only limited training in insonation, medical students have demonstrated greater diagnostic accuracy compared to the board-certified cardiologists performing traditional cardiac physical examination with stethoscopes [6].

While most modern medical school curricula continue to employ portable ultrasound units as the main teaching modality [7,8,9,10], the University of South Carolina introduced pocket ultrasound units to third-year medical students on Internal Medicine, fourth-year students on various electives, and during pediatrics and family medicine clerkships. However, none of the medical school programs have provided students with handheld units for their personal use in the first year and outside of a structured class or clerkship. We undertook this experiment of handheld units at the Mayo Medical School during the Human Anatomy course with the specific aim of allowing students to enhance their understanding of three-dimensional anatomy structures with self-directed learning, and to encourage their insonation skills throughout the first year.

## Methods

### The ultrasound curriculum for the First Year Medical Students

The ultrasound curriculum at the Mayo Medical School was originally incorporated into the first-year Gross Anatomy course a year prior to the introduction of handheld devices. The curriculum consisted of structured didactic sessions led by a large, interdisciplinary team including internists, endocrinologists, physiatrists, and physical therapists. Didactics were supplemented by review of online modules from Society of Ultrasound in Medical Education, as well as near-peer hands-on sessions supervised by teaching assistants (third year medical students) and various faculty members. Topics included basic introduction to ultrasound technology, abdominal vasculature and organ anatomy, long and short-axis cardiac anatomy, basic thyroid anatomy, and an introduction to imaging the carpal tunnel. Ultrasound instruction in different body parts coincided with learning of cadaveric anatomy (within the dissection hall) of the dedicated organ/area (Table 1).

**Table 1:** Ultrasound curriculum within Gross Anatomy Didactic block.


Week 1	Didactic + Hands-on introduction	Intro to Ultrasound – Knobology
Week 1	Hands-on workshop	Carpal Tunnel
Week 2	Hands-on workshop	Cardiac ultrasound: PSAX & PLAX
Week 3	Hands-on workshop	Abdominal Organs: Liver, Kidney & Gallbladder
Week 4	Hands-on workshop	Abdominal Vasculature: AAA, IVC
Week 5	Hands-on workshop	Neck US: Carotid, IJV & Thyroid
Week 6	OSPE Exams	Cardiac, Neck/Thyroid, Abdominal Organs, Abdominal Vessels


### Introduction of Handheld Ultrasound Equipment

In addition to the established ultrasound in anatomy curriculum, all first-year medical students (n = 53) in 2014 underwent training in the application of handheld ultrasound devices, conveniently called insonation, during the ultrasound near peer hands-on practice sessions. Towards the completion of the Anatomy course, each dissection team (4 students) received a handheld ultrasound device for the remainder of the academic year. The handheld personal devices were provided by Philips Healthcare (NUVIS, Philips, Buffalo, MN), and 13 handheld units were assigned to student teams. Each team received an ultrasound probe, a display tablet (Android; 19.85 cm × 12 cm × 10.5 cm) with a charger and a bottle of ultrasound gel. The entire assembly was lightweight, weighing 550 g, and provides high quality images (1280 × 800 IPS HD display). The transducer was a broadband curvilinear array with a frequency of 2–5MHz. The system was optimized for key clinical applications including: abdominal, vascular and pelvic imaging. The assembly for insonation was 510K cleared for diagnostic clinical use in the United States by the Food and Drug Administration.

The students were encouraged to use the device to scan themselves and each other in their free time, and to continue their exploration of anatomy throughout the ensuing academic blocks. Prior to participation, each student filled and signed a consent and disclaimer form developed in conjunction with the Mayo Clinic School of Medicine’s administration, which described at length the process in case of incidental findings. Student participation in the longitudinal component was encouraged but entirely voluntary.

### Evaluation of the practice of insonation

Electronic surveys using a 5-point Likert scale were developed by consensus among the ultrasound teaching faculty using previously employed and validated survey formats. These were sent out at quarterly intervals to all first-year medical students in order to assess their perceptions of the device as an educational tool for learning of ultrasound and human anatomy, ease and comfort level with the continuous use of the device, willingness to use ultrasound and perceived potential benefit of incorporation of insonation into the longitudinal medical school curricula, and likelihood of insonation on their selection of specialties for residency training. Survey questions are outlined in Table 2.

**Table 2:** Likert Scale Survey.

Survey Statements		Strongly disagree	Disagree	Neither agree nor disagree	Agree	Strongly Agree

1.	Having an ultrasound device at my personal disposal has improved my ability to perform ultrasound scans	1	2	3	4	5
2.	Having an ultrasound device at my personal disposal has improved my ability to interpret ultrasound images	1	2	3	4	5
3.	Having an ultrasound device at my personal disposal during the Anatomy course improved my understanding of the anatomical structures I imaged	1	2	3	4	5
4.	Having an ultrasound device at my personal disposal during medical school will influence my future choice of medical specialty	1	2	3	4	5
5.	I have had the opportunity to use the ultrasound device to improve my personal learning of human anatomy within the past 3 months	1	2	3	4	5
6.	I have had the opportunity to use the ultrasound device to augment my coursework at MMS within the past 3 months	1	2	3	4	5
7.	The NUVIS handheld ultrasound device is easy to use	1	2	3	4	5
8.	I am confident in my ability to use the NUVIS ultrasound device to create an image of an organ or other structures in the human body	1	2	3	4	5
9.	It would be beneficial if the use of ultrasound was implemented as a part of the longitudinal learning experience at MMS	1	2	3	4	5

The study protocol was approved by the Mayo Clinic institutional review board, in accordance with the Declaration of Helsinki.

### Data analysis

Mean Likert scale values and standard deviations (SD) for each electronic survey response to represent overall student agreement over different time points were obtained. Aiming to assess the student perception within the domains the responses were also presented as frequencies with percentages. Responses were summarized in 3 categories to reflect agreement (strongly agree/agree), neutral (neither agree nor disagree) and disagreement (disagree/strongly disagree) for all surveys. The analysis of variance (ANOVA) was used to for statistical comparisons, with two-tailed p-values < 0.05 considered statistically significant. All analyses were undertaken using JMP® statistical software, Version 13.0 (SAS Institute Inc., Cary, NC).

## Results

### Quantitative analyses

**Figure 1:**
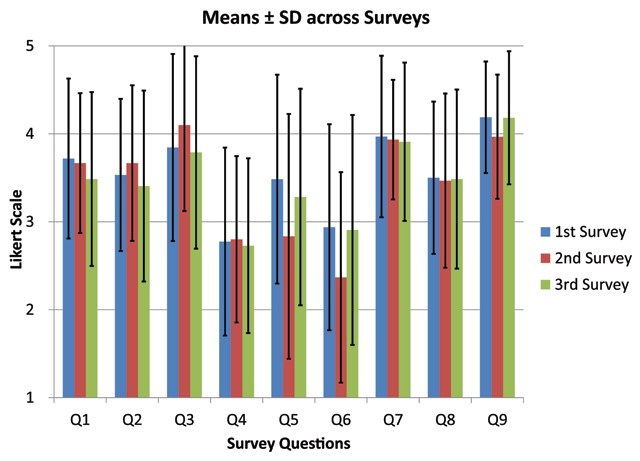
Mean Likert scale across survey timepoints.

Of the 3 surveys sent to the medical students over a period of twelve months, the response rate was 58.5% (n = 32) the first survey, 56% (n = 30) for the second survey, and 62.3% (n = 33) for the final survey, respectively, with an average response rate of 58.9%. Agreement was high across all survey questions (Figure 1), highest student agreement was observed when assessing enhanced understanding (Question 3, *mean ± SD 3.9 ± 1.01*), ease of use (Question 7, *3.9 ± 0.8*), and perceived benefit of incorporation on the curricula (Question 9, *4.1 ± 0.7*); the lowest agreement was reported for the effect on selection of medical specialty (Question 4, *2.8 ± 1.0*) and potential augmentation of coursework (Question 6, *2.7 ± 1.25*). Overall Survey responses across time points are presented in Figure 1. These results remained constant over follow-up surveys with the exception of the question assessing perceived benefit for further integration of insonation into the longitudinal curricula (p < 0.05) which increased significantly over time.

**Figure 2:**
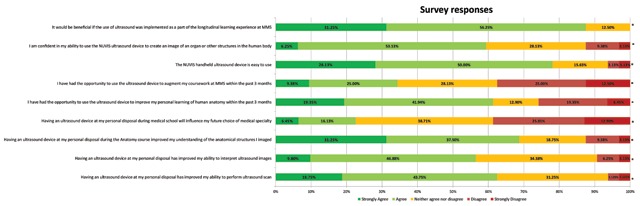
Level of agreement across survey questions at the baseline survey. * Denotes statistical significant differences across categories, p-value < 0.05.

At the baseline survey, overall agreement was observed for enhancement on performance (62.5%) and interpretation (56.3) of ultrasounds, understanding (68.8%) and learning of anatomy (61.3%), ease (78.1%), comfort (59.4%) and benefit of incorporation on ultrasound to the medical school curricula (p < 0.001 for all). Neutral (38.7%) or disagreement (38.7%) was observed when assessing the effect of integration of insonation within the medical curriculum on the choice of specialty for residency training (p < 0.01). Majority of disagreement was observed when assessing current access to the hand-held device (38.7%) (p < 0.001). Survey responses by categories are presented in Figure 2.

### Qualitative Analyses

At the end of the survey, students were encouraged to attach any additional comments they may have, pertaining to how the ultrasound experience could be improved. Of these comments, 4 major themes were identified. (1) a need for increased ultrasound instruction/curricular involvement outside of the Anatomy course, (2) limitations of the handheld device design and functionality, (3) time constraints due to other course work, and (4) challenges associated with having only one device shared among four students.

## Discussion

Of the nine questions addressed in the survey, those that showed the highest agreement among students suggest 3 resounding themes. First, students found that having an ultrasound device at their personal disposal during the Anatomy course improved their understanding of anatomical structures [11]. Second, they found the device easy to use, and thirdly, they strongly believed it to be beneficial for insonation training implemention as a part of the longitudinal learning experience across all school years. In addition, hands-on insonation training during gross anatomy course facilitated faster interpretation of ultrasound images, which could be linked to faster retrieval of anatomy knowledge needed for image analysis and facilitation of pattern recognition [12].

The most frequent comments provided by students referenced to the lack of opportunity to practice insonation in other academic blocks, accompanied by a desire for more structure and regularity in ultrasound education throughout the medical school years. These observations were verified as Question 6 received the lowest value of student agreement, that had queried if students had an adequate opportunity to use the ultrasound as a part of their academic courses over the preceding 3 months.

Several studies have examined the trainee perception of the value of ultrasound education during medical school and have consistently reported that ultrasound is a valuable tool which students anticipate using in their future careers [13]. The ultrasound education has witnessed a progressively increasing integration into the medical training [1,14,15,16,17,18,19,20,21,22]. However, there remains a lack of consensus on a uniform ultrasound curriculum in the medical school, along with several challenges [1,19]. Most institutions cite a lack of space in already over-crowded curriculum and lack of teaching and financial resources for equipment as the most significant barriers to integrating ultrasound education [1,19]. Most importantly, and confirmed in our study, the lack of student interest has not been found to be a barrier.

To overcome some of these reported and observed limitations and prior reported success with near peer teaching models, we trained and engaged interested senior medical students as hands on teachers (volunteer insonators) [20]. Students greatly valued this opportunity and appreciated an innovative approach to learning anatomy. Insonation sessions were effective in team building and allowed for a refreshing learning environment in otherwise fairly intense and challenging blocks in medical school teaching. The self-directed component was particularly welcomed by students as it built on flexibility without additional burden of classroom learning. We believe that introduction of cutting-edge ultrasound technology to students early in medical training would not only enhance their learning but also provide the benefit of developing a skill (or proficiency) to be potentially used later in clinical practice [12,21,22,23,24]. There is an increasing demonstration of the value of insonation for safe and high quality patient care in a variety of clinical settings. It is imperative that we prepare the next generation of medical students with up-to-date skills as the utility of point-of-care ultrasound, which depends on the experience and skills of the operator, and is affected by the availability of training and the cost of ultrasound devices [25].

### Limitations

Certain limitations to this study warrant discussion. The handheld device at the time of distribution contained only one low frequency, curvilinear probe appropriate for imaging largely abdominal structures. The higher frequency probes necessary for soft tissue and musculoskeletal imaging and continuous wave Doppler were not available. In addition, most courses at the Mayo medical school are currently structured as 7-week didactic blocks of accelerated material, thus making it not ideal for students to find time for self-directed learning. Lastly, due to limited resources, there was no scheduled face-to-face contact between anatomy faculty and students once the seven-week course had ended, thus limiting further teaching and feedback to electronic mail.

## Conclusions

These findings suggest that first-year medical students found the handheld ultrasound devices and insonation to be a valuable tool in learning and understanding of three-dimensional anatomy. More importantly, they were eager to engage in structured longitudinal learning through curricular inclusion of ultrasound rather than having to rely heavily on self-directed learning in other blocks.

## Data Accessibility Statement

The datasets generated during and/or analyzed during the current study are available from the corresponding author on reasonable request.
